# Evaluation and Validation of Reference Genes for qRT-PCR Normalization in *Frankliniella occidentalis* (Thysanoptera:Thripidae)

**DOI:** 10.1371/journal.pone.0111369

**Published:** 2014-10-30

**Authors:** Yu-Tao Zheng, Hong-Bo Li, Ming-Xing Lu, Yu-Zhou Du

**Affiliations:** 1 School of Horticulture and Plant Protection & Institute of Applied Entomology, Yangzhou University, Yangzhou, China; 2 Guizhou Institute of Plant Protection, Guiyang, China; USDA-ARS, United States of America

## Abstract

Quantitative real time PCR (qRT-PCR) has emerged as a reliable and reproducible technique for studying gene expression analysis. For accurate results, the normalization of data with reference genes is particularly essential. Once the transcriptome sequencing of *Frankliniella occidentalis* was completed, numerous unigenes were identified and annotated. Unfortunately, there are no studies on the stability of reference genes used in *F. occidentalis.* In this work, seven candidate reference genes, including *actin*, *18S rRNA*, *H3*, *tubulin*, *GAPDH*, *EF-1* and *RPL32*, were evaluated for their suitability as normalization genes under different experimental conditions using the statistical software programs BestKeeper, geNorm, Normfinder and the comparative ΔCt method. Because the rankings of the reference genes provided by each of the four programs were different, we chose a user-friendly web-based comprehensive tool RefFinder to get the final ranking. The result demonstrated that *EF-1* and *RPL32* displayed the most stable expression in different developmental stages; *RPL32* and *GAPDH* showed the most stable expression at high temperatures, while *18S* and *EF-1* exhibited the most stable expression at low temperatures. In this study, we validated the suitable reference genes in *F. occidentalis* for gene expression profiling under different experimental conditions. The choice of internal standard is very important in the normalization of the target gene expression levels, thus validating and selecting the best genes will help improve the quality of gene expression data of *F. occidentalis*. What is more, these validated reference genes could serve as the basis for the selection of candidate reference genes in other insects.

## Introduction

Quantitative measurements of gene expression are increasingly important for understanding biological processes. Knowledge of a gene’s expression profile can provide evidence about its regulation and function [1]. Because of its accuracy, high sensitivity and reproducibility, quantitative real-time PCR (qRT-PCR) [2], is generally considered the best method for use in a variety of experimental and clinical conditions to analyze gene expression [3]–[4]. For accurate gene quantification analysis, normalization of qRT-PCR data is essential in order to eliminate variations in initial mRNA samples, sampling methods, reverse-transcription, and amplification efficiencies among different samples [5]–[6]. The most common normalization method is to relate mRNA levels of target genes to reference genes whose expression levels are highly stable in living organisms during various phases of development or under different environmental/experimental conditions [7].

Many genes involved in basic, ubiquitous cellular functions are housekeeping genes [8], such as *actin*, *18S rRNA*, and glyceraldehyde-3-phosphate dehydrogenase (*GAPDH*) are commonly used as reference genes due to presumed uniform levels of expression. It is believed that there are few fluctuations in the transcription level of these genes compared to other genes [7], [9]. However, many studies have shown that the expression levels of housekeeping genes do not always remain constant, thereby compromising their use as stable internal standards [10]. Moreover, errors in expression data of up to 20-fold have been demonstrated in instances where a single reference gene was used [11]. Therefore, it is critical to validate the expression stability of reference genes under different experimental conditions before using them for normalization.

The western flower thrips, *Frankliniella occidentalis*, is a notorious invasive species in many countries, causing serious economic damage to vegetables and ornamental plants [12]. It is both a direct pest of crops and an efficient vector of plant viruses such as tomato-spotted wilt virus [13]. In China, the western flower thrips was first recorded in Beijing in 2003 [14], and a recent investigation reported that it has spread to more than 10 provinces [15]. Sequencing of the western flower thrips genome was recently included in the 5,000 insect genome initiative, reflecting the rising importance of this invasive pest (http://arthropodgenomes.org/wiki/i5K). Meanwhile, rapid progress has been made in the sequencing of western flower thrips by means of transcriptome analysis [16], [17]. These data provide comprehensive gene expression information at the transcriptional level that may facilitate our understanding of the molecular mechanisms underlying various physiological aspects, including the development and response of western flower thrips to environmental stress [18].

To examine the changes of gene expression in *F. occidentalis*, Li *et al.* used *18S rRNA,* the most frequently used reference gene in qRT-PCR analyses [19]. Because reference genes for *F. occidentalis* were selected without a companion validation study to evaluate their suitability under specific experimental conditions, we have evaluated the stability and performance of the following seven candidate reference genes: 18S ribosomal RNA (*18S rRNA*), β-*actin* (*actin*), *tubulin*, elongation factor-1 alpha (*EF-1*), glyceraldehyde-3-phosphate dehydrogenase (*GAPDH*), histone 3 (*H3*) and ribosomal protein L32 (*RPL32*) under different experimental conditions (developmental stage, high temperature, and low temperature). The reference genes identified here will help future research to more precisely assess gene expression, to better understand the functionality of target genes and to elucidate specific molecular mechanisms underpinning the physiological processes in *F. occidentalis*.

## Materials and Methods

### Insects

*F. occidentalis* was originally collected in Hangzhou, China in 2008 from a field maintained by our group and reared in the laboratory as described by Li *et al*. [20]. Individuals collected at the first day of each developmental stage were used in the experiment. We declare that no specific permissions were required for these activities and that the field studies did not involve endangered or protected species.

### Samples from different developmental stages

*F. occidentalis* from different developmental stages were collected separately and pooled by stage for gene analysis with more individuals pooled from the earlier life stages: 1st instar nymphs (300 per pool), 2nd instar nymphs (200 per pool), pupae (200 per pool) and adults (150 per pool). Four replicate pools for each stage were then collected for RNA extraction.

### High temperature-induced stress

Each pool of 200 2nd instar nymphs were exposed to temperatures of 31, 33, 35, 37, 39 and 41°C for 2 h in a glass tube placed in a metal bath. A pool of nymphs were held at 26°C as a control. Four samples from each temperature were then collected for RNA extraction.

### Low temperature-induced stress

Each group of 200 2nd instar nymphs were exposed to temperatures of 2, 0, −2, −4, −6, −8 and −10°C for 2 h in a glass tube placed in a metal bath. A pool of nymphs were held at 26°C as a control. Four samples from each temperature were then collected for RNA extraction.

### Selection of gene sequences and primer design

Primer sequences and the associated amplicons characteristics for seven commonly used reference genes are summarized in Table 1. Based on reference genes described in the literature, the NCBI database (http://www.ncbi.nlm.nih.gov) was searched for homologous *F. occidentalis* sequences: *18S*, *actin*, *EF-1*, *RPL32*, *tubulin*. And *GAPDH* and *H3* sequences were amplified based on homologies with the hemipteran insects. All gene sequences have been submitted to GenBank. The Primer Premier 5 software (http://www.premierbiosoft.com/primerdesign/index.html) was used to design the primers. The parameters in Primer 5 were set as follows: amplicon length 70–150 bp, melting temperature 58–62°C, primer lengths 20–26 bp, and GC content 40–60%.

**Table 1:** Primer sequences and amplicon characteristics of the candidate reference genes.

Gene	Primer Name	Primer Sequence (5′-3′)	Size^a^(bp)	Tm^b^(°C)	E^c^(%)	R^2d^
actin	actin-F	ATGTTCCAGCCGTCCTTCTT	157	55.6	100.0	0.999
	actin-R	TTCTGTCAGCAATGCCAGGGTA				
18S	18S-F	AACACGGGAAACCTCACCA	116	55.4	108.9	0.997
	18S-R	CAGACAAATCGCTCCACCAA				
H3	H3-F	TCAAACAGACCAACGAGGTAAGC	96	55.9	106.2	0.995
	H3-R	GATCGCCCAGGACTTCAAAA				
tubulin	tubulin -F	CTGAGATGACAGGGGCATAAGT	129	60.6	98.3	0.996
	tubulin -R	TGTCTTCCATCACGGCTTCC				
GAPDH	GAPDH-F	AAGGGTGCTCAGGTTGTTGCT	89	56.5	104.4	0.990
	GAPDH-R	CGACCGTGGGTGGAGTCATAT				
EF-1	EF-1-F	TCAAGGAACTGCGTCGTGGAT	130	58.6	95.4	0.999
	EF-1-R	ACAGGGGTGTAGCCGTTAGAG				
RPL32	RPL32-F	CAACATCGGTTATGGAAGCA	141	55.0	100.1	0.998
	RPL32-R	ACAGCGTGGGCAATTTCAGC				
Hop	Hop-F	CCTTTCCCTGGTGCTGGTT	86	57.1	95.9	0.992
	Hop-R	GCTCTTGTTTGCGGGTTGTT				

a Length of the amplicon; ^b^melt temperature; ^c^Real-time qPCR efficiency (calculated from the standard curve); ^d^Regression coefficient.

### Quantitative Real-time PCR analysis

Total RNA was extracted using the SV Total RNA isolation system (Promega Z3100 (Promega, USA)), followed by DNase treatment to eliminate DNA contamination. The integrity of the RNA in all samples was verified by comparing the ribosomal RNA bands in ethidium bromide-stained gels. RNA sample purity was estimated using spectrophotometric measurements at 260 and 280 nm (Eppendorf Biophotometer plus (Eppendorf, Germany)). The OD260/280 of all samples was 1.8–2.2. Real-time PCR reactions were performed in a 20 µl total reaction volume comprised of 10 µl of iTaq universal SYBR Green supermix(2x) (Bio-Rad Laboratories Inc., USA), 1 µl of each gene specific primer (Table 1), 2 µl of cDNA templates, and 6 µl of PCR-grade water. Reactions were carried out on a CFX-96 real-time PCR system (Bio-Rad Laboratories Inc., USA). The efficiencies of the target and reference genes were similar [5], [21]. The PCR parameters for all genes were as follows: 95°C for 3 min, 40 cycles of 95°C for 5 sec, 30 sec at the Tm value of primer pairs (Table 1) with melting curve analysis performed to determine the specificity of PCR products. Every treatment included four replicates, and each reaction was run in triplicate.

### Evaluation of target gene expression

*Hop*, also known as stress-induced protein-1, is a co-chaperone that usually acts as an adaptor, mediating the association of molecular chaperones *Hsp90* and *Hsp70* [19]. *Hop* was used as a target gene to evaluate the candidate reference genes. Relative quantification of *Hop* in different samples was conducted according to threshold cycle (Ct) value based on 2^−ΔΔCt^ method.

### Statistical analysis

Expression levels were determined as the number of cycles needed for the amplification to reach a fixed threshold in the exponential phase of the PCR reaction [22]. This threshold was set at 400 for all genes to determine the Ct values. The stability of candidate genes was evaluated by three commonly used software tools: BestKeeper (http://www.wzw.tum.de/genequantification/bestkeeper.html) [23], [24], geNorm version 3.5 (http://medgen.ugent.be/~jvdesomp/genorm/index.php) [8], and NormFinder-0953 (http://www.mdl.dk/publicationsnormfinder.htm) [25]. Finally, we compared and ranked the tested candidates based on a web-based analysis tool, RefFinder (http://www.leonxie.com/referencegene.php?type=reference), including the comparative ΔCt method, to compare and rank the tested candidate reference genes [26]. The Bestkeeper index is based on the raw data and PCR amplification efficiency to determine the best-suited standards and combines them to create an index. Lower index scores denote greater transcriptional stability and thus better suitability as a reference gene. Ct values were converted into relative quantities and imported into the geNorm and NormFinder software programs. The geNorm algorithm first calculates an expression stability value (M) for each gene and then compares the pair-wise variation (V) of this gene with the others. The value of Vn/Vn+1 indicates the pairwise variation between two sequential normalization factors and determines the optimal number of reference genes required for accurate normalization [27]. Using microarray data as a training set for the algorithm, a threshold of V <0.15 was suggested for valid normalization [8]. NormFinder uses a model-based approach to estimate expression variation in the selection of suitable reference genes [25]. Genes with the lowest values are the most stable. Based on the rankings from each program, RefFinder assigns an appropriate weight to an individual gene and calculates the geometric mean of their weights for the overall final ranking. Multiple comparisons of Ct values were performed by analysis of variance (One-Way ANOVA) followed by Tukey s-b(k) at the 95% confidence level using PASW Statistics version 18 (IBM Corp., Somers, NY).

## Results

### Total RNA Quality and PCR Amplification Efficiencies

The concentration and purity levels of total RNA isolated from different samples were determined using the Eppendorf Biophotometer plus. The A260/A280 ratios ranged from 1.80 to 2.20 for RNA samples, indicating a high purity of total RNA for all samples. The integrity of all total RNA samples was confirmed using 1.0% agarose gel electrophoresis. For each of the primer pairs, single peak qRT-PCR melting curves suggested that each of the primer pairs amplified a unique product. A standard curve was generated for each gene, using a ten-fold serial dilution of the pooled cDNAs. The correlation coefficient and PCR efficiency for each standard curve are shown in Table 1. The PCR efficiency of the seven candidate reference genes and one target gene were similar, ranging from the lowest for *EF-1* (95.4%) to the highest for *18S* (108.9%). Linear regression coefficients (R^2^) for all eight genes were ≥0.990. The melting temperatures (Tm) of all PCR products ranged from 55.0°C for *RPL32* to 60.6°C for *tubulin*.

### Expression profile of candidate reference genes

**Figure 1:**
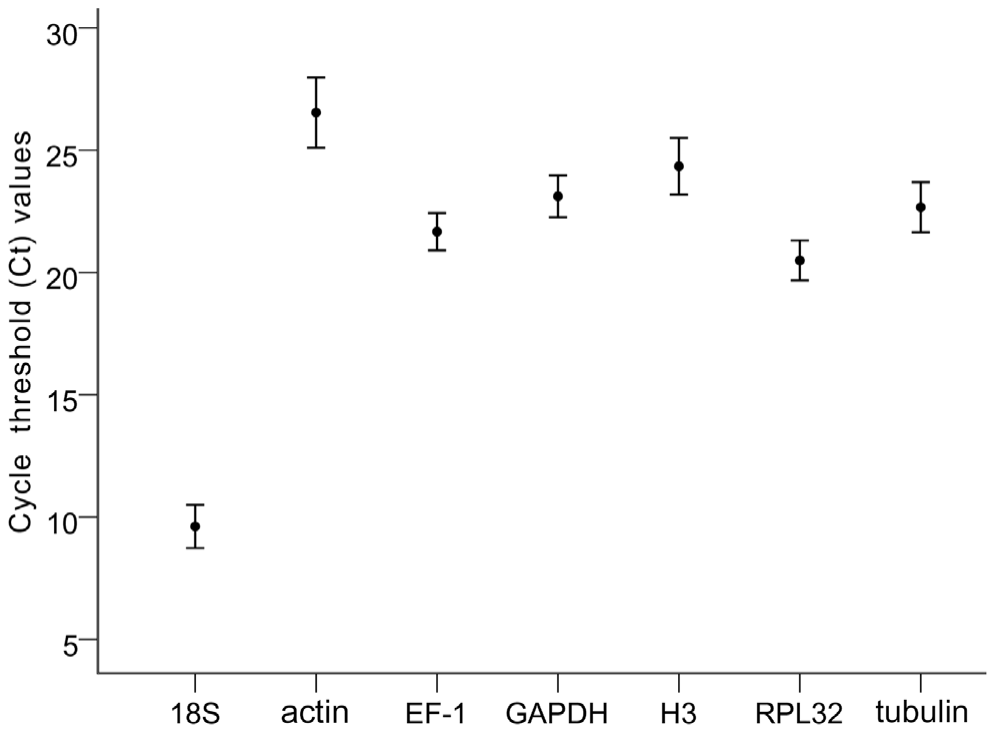
Expression levels of candidate reference genes in different samples. The black dot indicates the mean of duplicate samples, and the bars indicate the standard deviation of the mean.

Expression levels were determined as the number of cycles needed for amplification to reach a fixed threshold in the exponential phase of the qPCR reaction [21]. Except for *18S*, the gene expression analysis of all seven candidate reference genes displayed a narrow range of mean Ct values across all experimental samples (Fig. 1). The mean Ct values of the seven reference genes varied from 9.62 (*18S*) to 26.54 (*actin*). The *18S* gene showed the most abundant expression levels, followed by *RPL32* (mean Ct 20.49), *EF-1*(mean Ct 21.67), *tubulin* (mean Ct 22.67), *GAPDH* (mean Ct 23.12), and *H3* (mean Ct 24.35). The smallest Ct variation among the experimental samples was *EF-1* with a value of 4.01, while *actin* had the highest Ct variation with a value of 7.74. All candidate genes exhibited relatively small variation in Ct values.

### Analysis of gene expression stability

#### Developmental stages

**Figure 2:**
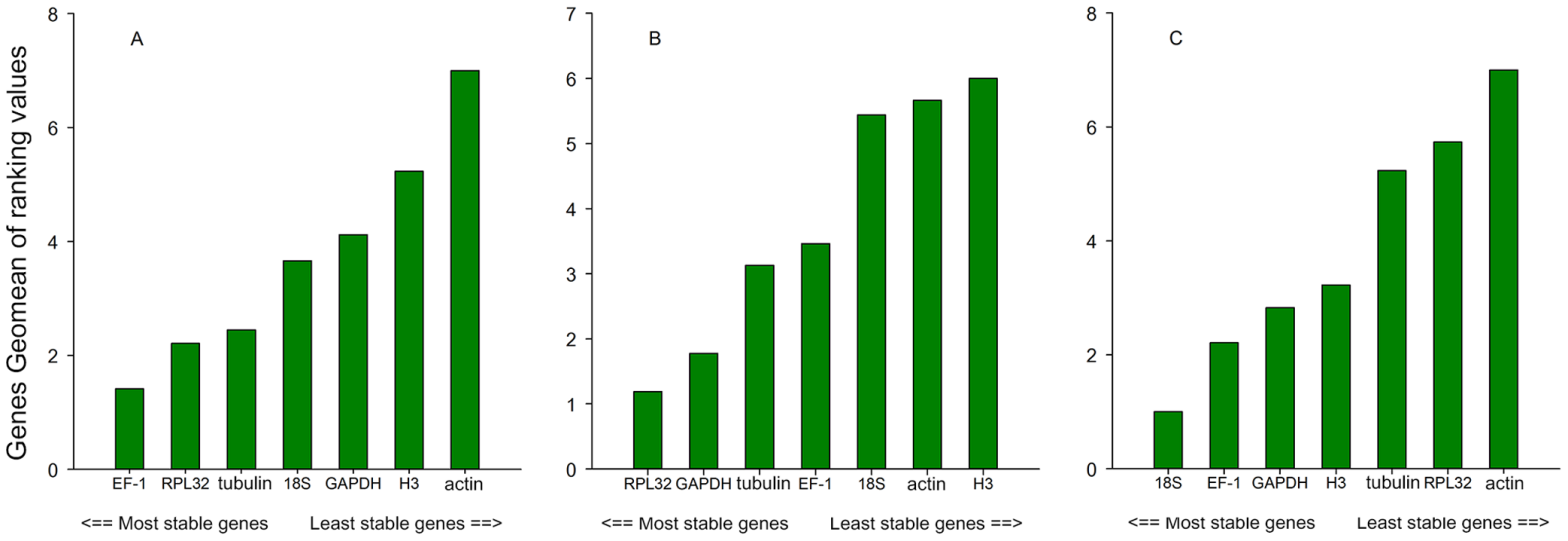
Expression stability of the candidate reference genes as calculated by the Geomean method of RefFinder. A lower Geomean ranking indicates more stable expression. Expression stability of reference genes in the following samples: A) different developmental stages of *Frankliniella occidentalis*; B) *F. occidentalis* exposed to high temperatures; C) *F. occidentalis* exposed to low temperatures.

**Figure 3:**
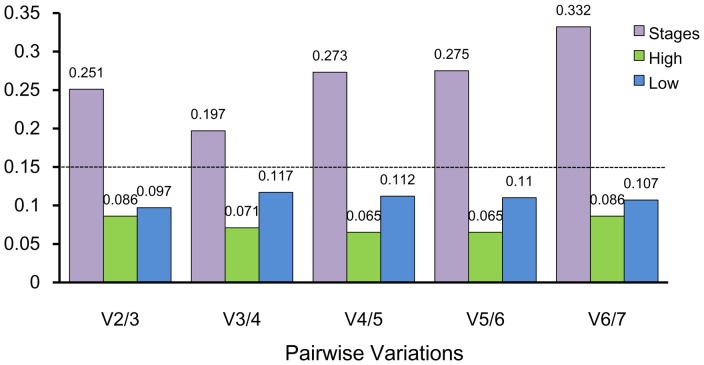
Optimal number of reference genes for normalization in *Frankliniella occidentalis.* The pairwise variation (Vn/Vn+1) was analyzed between normalization factors NFn and NFn+1 by geNorm program to determine the optimal number of reference genes. Values <0.15 indicate that additional genes are not required for the normalization of gene expression.

The stability rankings generated by the comparative ΔCt method were largely similar with the results obtained from geNorm. However, the most stable genes as ranked by BestKeeper and NormFinder analysis were different from the results generated by geNorm and the comparative ΔCt method. The geNorm and comparative ΔCt method identified *actin* as the least stable gene and *EF-1* as the most stable gene. In contrast, BestKeeper and NormFinder identified *EF-1* as the second most stable gene. According to the results of RefFinder, the stability ranking from the most to the least stable in the developmental stages was *EF-1*>*RPL32*> *tubulin* >*18S*>*GAPDH*>*H3*>*actin* (Fig. 2). However, GeNorm analysis revealed that all the pair-wise variation values were above the proposed 0.15 cut-off (Fig. 3). These results indicate that normalization with three stable reference genes (*EF-1, RPL32, tubulin* ) was required (as suggested by the geNorm manual).

#### High temperature

Most of the programs with the exception of BestKeeper identified *RPL32* and *GAPDH* as the most stable genes and *actin* as the least stable gene. However, *RPL32* was also idenitified as the most stable gene, but *GAPDH* ranked fifth in BestKeeper (Table 2). According to the results of RefFinder, the stability ranking from the most stable to the least stable gene in high temperature-stressed samples was *RPL32*>*GAPDH*> *tubulin* >*EF-1*>*18S*>*actin*>*H3* (Fig. 2). GeNorm analysis revealed that all the pairwise variation values were below the proposed 0.15 cut-off value (Fig. 3). According to geNorm, two reference genes (*RPL32* and *GAPDH*) should be required for a suitable normalization in the high temperature-stressed samples (Fig. 2).

**Table 2:** Ranking order of the candidate reference genes of *Frankliniella occidentalis* under different experimental conditions.

Conditions	Rank	Delta Ct		BestKeeper		NormFinder		geNorm	
		Gene name	Standard deviation	Genename	Standard deviation	Gene name	Stability value	Gene name	Stability value
**Developmental stages**	1	EF-1	1.193	18S	0.741	tubulin	0.316	EF-1/RPL32	0.116
	2	RPL32	1.213	EF-1	0.755	EF-1	0.356		
	3	tubulin	1.317	tubulin	0.762	RPL32	0.428	GAPDH	0.542
	4	GAPDH	1.371	RPL32	0.818	GAPDH	0.562	tubulin	0.698
	5	H3	1.785	H3	0.956	18S	0.945	H3	0.985
	6	18S	1.820	GAPDH	1.026	H3	1.019	18S	1.249
	7	actin	2.455	actin	1.458	actin	1.587	actin	1.593
**High temperature**	1	GAPDH	0.359	RPL32	0.360	RPL32	0.102	GAPDH/RPL32	0.278
	2	RPL32	0.377	tubulin	0.385	GAPDH	0.103		
	3	EF-1	0.387	actin	0.401	tubulin	0.130	EF-1	0.288
	4	tubulin	0.391	EF-1	0.455	EF-1	0.149	tubulin	0.308
	5	18S	0.472	GAPDH	0.491	18S	0.266	18S	0.311
	6	H3	0.494	H3	0.609	H3	0.277	H3	0.396
	7	actin	0.636	18S	0.629	actin	0.409	actin	0.455
**Low temperature**	1	18S	0.494	18S	0.183	18S	0.087	GAPDH/18S	0.276
	2	EF-1	0.541	EF-1	0.246	EF-1	0.203		
	3	H3	0.577	H3	0.307	H3	0.215	EF-1	0.307
	4	GAPDH	0.595	GAPDH	0.324	GAPDH	0.295	H3	0.401
	5	tubulin	0.703	tubulin	0.365	tubulin	0.382	RPL32	0.484
	6	RPL32	0.730	RPL32	0.429	RPL32	0.419	tubulin	0.562
	7	actin	0.830	actin	0.563	actin	0.504	actin	0.639

The expression stability was also measured using the **Δ**Ct method, BestKeeper, NormFinder, and geNorm and ranked from the most stable to the least stable.

#### Low temperature

The overall stability ranking generated by the comparative ΔCt method was largely similar with the results obtained by NormFinder and BestKeeper. All four programs identified *18S* as the most stable gene and *actin* as the least stable gene in low temperature-stressed samples (Table 2). RefFinder analysis ranked gene stability as *18S*>*EF-1*>*GAPDH*>*H3*> *tubulin* >*RPL32*>*actin*. GeNorm analysis revealed that the pairwise variation values were below the proposed 0.15 cut-off value (Fig. 3). According to geNorm, two reference genes (*18S* and *EF-1*) should be required for a suitable normalization in the low temperature-stressed samples (Fig. 2).

### Validation of reference gene selection

To assess the validity of selected reference genes, the relative expression of the target gene *Hop* was estimated for different experimental conditions. Target expression analyses further showed that differences in quantification were detected when normalizing with arbitrary reference genes relative to the best reference genes. We compared the mRNA transcript level of *Hop* when using two best reference genes *RPL32* and *GAPDH* for high temperature-stressed samples, *18S* and *EF-1* for low temperature-stressed samples and the three best reference genes (*EF-1*, *RPL32* and *tubulin*) for the different developmental stages along with the most unstable gene *actin,* which is usually recommended for normalization by RefFinder.

**Figure 4:**
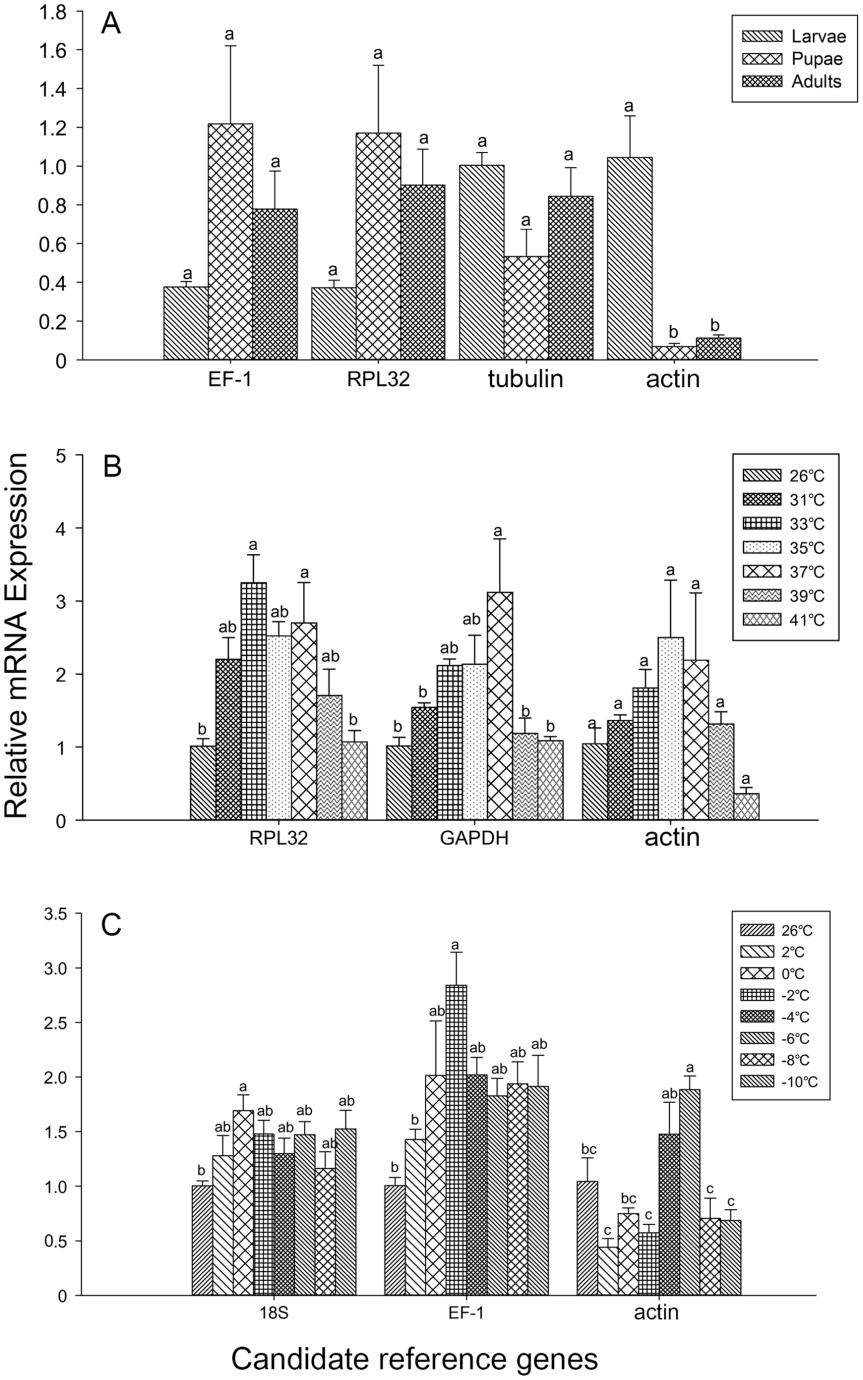
Validation of reference gene selection. A) Relative expression levels of *Hop* in different developmental stages; B) Exposed to high temperatures; C) Exposed to low temperatures (*P*<0.05, Tukey s-b(k); n = 4).

When using arbitrary genes to determine the relative expression levels of target genes in different samples the results can differ. For example, when using the most unstable gene for normalization, *Hop* transcript levels were higher in the nymphal stage compared to adult and pupae in the different development stages. However, after the best reference gene or the recommended two most stable references were used to normalize, no evident difference was detected (Fig. 4A). Similar results also occurred when calculating the relative expression levels of *Hop* after normalization with the unstable reference genes among the samples exposed to low temperatures (Fig. 4C). Therefore, it is important to determine the optimal reference genes for accurate normalization of qRT-PCR data, especially when differences in expression levels are subtle, as arbitrary selection of reference genes may decrease the accuracy of determining target gene expression. For example, the relative expression level of *Hop* showed no significant differences among the samples exposed to high temperatures when calculated using *actin* as the reference gene; however, the expression was significantly different when normalized by *RPL32* and *GAPDH* (Fig. 4B). Therefore, in order to obtain accurate expression data, the expression stability of putative reference genes needs to be verified before each qRT-PCR experiment.

## Discussion

Western flower thrips is considered one of the most economically important pests of agricultural crops worldwide [28], [29]. Due to these thrips small size and secluded behavior, there is no effective way to control them. Previous studies concerning the transcriptome sequencing for *F. occidentalis* have been conducted, and numerous unigenes have been annotated according to their putative functional categories. While these sequence and putative functional data serve as a valuable resource for the control of this pest, it will be essential to validate the gene functions, including discovering their expression profile [16], [17]. qRT-PCR is a sensitive, accurate and operable method to comparatively quantify transcript expression levels and profile gene expression levels [5], [30], [31]. Several studies on reference gene validation have emphasized that multiple internal genes must be evaluated in order to improve the accuracy of the qRT-PCR analysis and interpretation of gene expression [8], [32], [33]. Because few reference genes have been systematically evaluated in *F. occidentalis*, we identified suitable reference genes from different experimental conditions.

This study was conducted to identify the optimal reference genes for gene expression analyses of *F. occidentalis* from different developmental stages and temperature stress conditions. Seven candidate reference genes were analyzed by qRT-PCR. To our knowledge, this is the first study to evaluate the expression stability of different candidate reference genes for qRT-PCR in *F. occidentalis*. As expected, the most stable reference genes varied across the different experimental conditions (Fig. 2). Similar to *Schistocerca gregaria* [1], EF-1 and RPL32 displayed the most stable expression in development. RPL32 and GAPDH were the most stable under high temperature stress conditions, while *18S* and *EF-1* exhibited the most stable under low temperature stress conditions. These results indicate that the stability of reference genes expression in *F. occidentalis* needs to be determined for each experimental condition.

Previously, the structural protein actin, which is expressed at moderately abundant levels in most cell types, has been considered an ideal reference gene. *actin* was found to be one of the most suitable reference genes in studies of gene expression in *Apis mellifera* [34], *Schistocerca gregaria* [1], *Drosophila melanogaster* [35], *Plutella xylostella* and *Chilo suppressalis* [36], *Chortoicetes terminifera* [37] and *Diuraphis noxia* [38]. However, in our study, *actin* ranked as one of the least stable genes in almost all of the experimental conditions, *actin* is not a suitable reference gene for *F. occidentalis* under those experimental conditions. This result is consistent with previous studies that *actin* is not stable in *Nilaparvata lugens* [18] or *Solenopsis invicta* [39].

Although only a single reference gene with high expression stability may be appropriate for normalization of gene expression under some experimental conditions, in most experimental conditions two or more reference genes are required for accurate and reliable results [8]. In this study, we determined the optimal number of reference genes under different experimental conditions as calculated by geNorm, and calculated the two sequential normalization factors (NF_n_ and NF_n_+1) needed to determine the minimum number of reference genes. Vandesompele *et al*. [8] proposed 0.15 as a cut-off value for V, below this value the inclusion of additional control genes is not required. In our studies, V values in high temperatures and low temperatures were both lower than the threshold value (0.15); therefore, we suggest that the two best reference genes be used to obtain accurate and reliable results under these conditions. Since, V values for the developmental stages were higher than the threshold value (0.15), we suggest that the three best reference genes be used for these conditions as the 0.15 recommended by geNorm (2007) is a suggested starting point.

Previous reference gene validation studies combined high and low temperature treatments, collectively referring to them as temperature treatment [18], [27], [40], [41]. Interestingly, we found that the gene rankings for high and low temperature were different (Fig. 2). The most stable reference genes under high temperature-stress conditions were *RPL32* and *GAPDH*, whereas *RPL32* was one of the least stable reference genes under low temperature-stress conditions (Fig. 2). In contrast, the most stable reference genes under low temperature stress were 18S and EF-1. In general, expression of insect genes may vary under different temperature stesses. For example, *hsp19.7* and *hsp20.7* of *Spodoptera litura* responded only to heat treatment, not cold stress, while *hsp21.5* of *Chilo suppressalis* only responded to cold stress [42], [43]. Therefore, we believe that it is more reasonable to validate reference genes separately for high and low temperature.

Although the rankings of the different reference genes provided by three of the programs (BestKeeper, NormFinder and geNorm) were similar, our results showed that the determination of the most appropriate reference gene can vary depending on the software used (Table 2). For the developmental stages, geNorm recommended the use of *EF-1* and *RPL32*, NormFinder recommended *tubulin* and BestKeeper recommended *18S*. To determine which was the best reference gene, we chose a user-friendly web-based comprehensive tool called RefFinder. Based on the rankings from the computational programs (geNorm, Normfinder, BestKeeper, and the comparative ΔCt method), it assigned an appropriate weight to an individual gene and calculated the geometric mean of their weights to generate an overall final ranking. To validate the RefFinder-determined results, the expression of *Hop* was investigated (Fig. 2). Previous studies have demonstrated that *Hop* is significantly upregulated under high temperature stress conditions [21]. Compared to the more suitable reference genes, the use of *actin* as an internal control resulted in erroneous expression profiles (Fig. 4). The quantification of *Hop* using different control genes showed that the choice of the internal standard is very important in the normalization of the target gene expression levels.

## Conclusions

In conclusion, this work is the first study aimed at validating a set of candidate reference genes for gene expression normalization using qRT-PCR in *F. occidentalis*. In the present research, seven candidate reference genes were identified. We concluded that *EF-1* and *RPL32* are the most suitable reference genes for the analysis of developmental stages, *RPL32* and *GAPDH* are the most suitable reference genes under high temperature stress, and *18S* and *EF-1* are the most suitable reference genes under low temperature stress. These results may constitute a starting point for selecting reference genes in the future for more accurate normalization under other experimental conditions in *F. occidentalis.*

## References

[1] Van Hiel MB, Van Wielendaele P, Temmerman L, Van Soest S, Vuerinckx K, et al (2009) Identification and validation of housekeeping genes in brains of the desert locust *Schistocerca gregaria* under different developmental conditions. BMC Mol Biol 10(1): 56. 19508726 10.1186/1471-2199-10-56 PMC2700112

[2] Bustin SA (2000) Absolute quantification of mRNA using real-time reverse transcription polymerase chain reaction assays. J Mol Endocrinol 25(2): 169–193. 11013345 10.1677/jme.0.0250169

[3] Bustin SA, Benes V, Nolan T, Pfaffl MW (2005) Quantitative real-time RT-PCR-a perspective. J Mol Endocrinol 34: 597–601. 15956331 10.1677/jme.1.01755

[4] Citri A, Pang ZP, Südhof TC, Wernig M, Malenka RC (2012) Comprehensive qRT-PCR profiling of gene expression in single neuronal cells. Nat Protoc. 7: 118–117. 10.1038/nprot.2011.430 PMC4377330 22193304

[5] Nolan T, Hands RE, Bustin SA (2006) Quantification of mRNA using real-time RT-PCR. Nat Protoc. 1: 1559–1582. 10.1038/nprot.2006.236 17406449

[6] Lu Y, Yuan M, Gao X, Kang T, Zhan S, et al. (2013). Identification and validation of reference genes for gene expression analysis using quantitative PCR in *Spodoptera litura* (Lepidoptera: Noctuidae). PLoS ONE, 8(7), e68059. 10.1371/journal.pone.0068059 PMC3706614 23874494

[7] Thellin O, Zorzi W, Lakaye B, De BB, Coumans B, et al (1999) Housekeeping genes as internal standards: use and limits. J Biotechnol 75: 291–295. 10617337 10.1016/s0168-1656(99)00163-7

[8] Vandesompele J, De PK, Pattyn F, Poppe B, Van RN, et al (2002) Accurate normalization of real-time quantitative RT-PCR data by geometric averaging of multiple internal control genes. Genome Biol. 3: 1–11. 10.1186/gb-2002-3-7-research0034 PMC126239 12184808

[9] Provenzano M, Mocellin S (2007) Complementary techniques: validation of gene expression data by quantitative real-time PCR. Eurekah Bioscience 2(6): 510–513. 10.1007/978-0-387-39978-2_7 17265717

[10] Ruan WJ, Lai MD (2007) *Actin*, a reliable marker of internal control ? Clinica Chimica Acta 385(1–2): 1–5. 10.1016/j.cca.2007.07.003 17698053

[11] Warrington JA, Nair A, Mahadevappa M, Tsyganskaya M (2000) Comparison of human adult and fetal expression and identification of 535 housekeeping/maintenance genes. Physiol Genomics 2: 143–147. 11015593 10.1152/physiolgenomics.2000.2.3.143

[12] Reitz SR (2009) Biology and ecology of the western flower thrips (Thysanoptera: Thripidae): the making of a pest. Fla. Entomol 92: 7–13.

[13] Wijkamp I, Almarya N, Goldbach R, Peters D (1995) Distinct levels of specificity in thrips transmission of tospoviruses. Phytopathology 85: 1069–1074.

[14] Zhang YJ, Wu QJ, Xu BY, Zhu GR (2003) Invasive insect pest, western flower thrips, brought out and damaged in Beijing. Plant Prot 29: 58–59.

[15] Chen XL, Yuan LZ, Du YZ, Zhang YJ, Wang JJ (2011) Cross-resistance and biochemical mechanisms of abamectin resistance in the western flower thrips, *Frankliniella occidentalis*, Pestic. Biochem. Physiol 101: 34–38.

[16] Zhang Z, Zhang P, Li W, Zhang J, Huang F, et al (2013) De novo transcriptome sequencing in *Frankliniella occidentalis* to identify genes involved in plant virus transmission and insecticide resistance. Genomics 101(5): 296–305. 23434629 10.1016/j.ygeno.2013.02.005

[17] Rotenberg D, Whitfield AE (2010) Analysis of expressed sequence tags for *Frankliniella occidentalis*, the western flower thrips. Insect Mol Biol 19(4): 537–551. 20522119 10.1111/j.1365-2583.2010.01012.x

[18] Yuan M, Lu YH, Zhu X, Wan H, Muhammad S, et al (2014) Selection and evaluation of potential reference genes for gene expression analysis in the Brown Planthopper, *Nilaparvata lugens* (Hemiptera: Delphacidae) using reverse-Transcription Quantitative PCR. PLoS ONE 9(1): e86503. 24466124 10.1371/journal.pone.0086503 PMC3900570

[19] Li HB, Du YZ (2013) Molecular cloning and characterization of an Hsp90/70 organizing protein gene from *Frankliniella occidentalis* (Insecta: Thysanoptera, Thripidae). Gene 520(2): 148–155. 23458874 10.1016/j.gene.2013.02.026

[20] Li HB, Shi L, Lu MX, Wang JJ, Du YZ (2011) Thermal tolerance of *Frankliniella occidentalis*: effects of temperature, exposure time and gender. Therm. Biol 36: 437–442.

[21] Bustin SA, Benes V, Garson JA, Hellemans J, Huggett J, et al.. (2009). The MIQE guidelines: minimum information for publication of quantitative real-time PCR experiments. Clinical chemistry 55(4), 611–622. 10.1373/clinchem.2008.112797 19246619

[22] Walker NJ (2002) A technique whose time has come. Science 296: 557–559. 11964485 10.1126/science.296.5567.557

[23] Pfaffl MW (2004) Quantification strategies in real-time PCR. AZ of quantitative PCR 1: 89–113.

[24] Pfaffl MW, Tichopad A, Prgomet C, Neuvians TP (2004) Determination of stable housekeeping genes, differentially regulated target genes and sample integrity: BestKeeper-Excel-based tool using pair-wise correlations. Biotechnol Lett 26: 509–515. 15127793 10.1023/b:bile.0000019559.84305.47

[25] Andersen CL, Jensen JL, Ørntoft TF (2004) Normalization of real-time quantitative reverse transcription-PCR data: a model-based variance estimation approach to identify genes suited for normalization, applied to bladder and colon cancer data sets. Cancer Res 64(15): 5245–5250. 15289330 10.1158/0008-5472.CAN-04-0496

[26] Xie F, Sun G, Stiller JW, Zhang B (2011) Genome-wide functional analysis of the cotton transcriptome by creating an integrated EST database. PLoS ONE 6: e26980. 22087239 10.1371/journal.pone.0026980 PMC3210780

[27] Li R, Xie W, Wang S, Wu Q, Yang N, et al (2013) Reference gene selection for qRT-PCR analysis in the sweet potato whitefly, *Bemisia tabaci* (Hemiptera: Aleyrodidae). PLoS ONE. 8(1): e53006. 10.1371/journal.pone.0053006 PMC3540095 23308130

[28] Bielza P, Quinto V, Contreras J, Torné M, Martín A (2007) Espinosa Resistance to spinosad in the Western flower thrips, *Frankliniella occidentalis* (Pergande), in greenhouses of south-eastern Spain. Pest Manag Sci 63: 682–687. 17487830 10.1002/ps.1388

[29] Tommasini MG, Maini S (1995) *Frankliniella occidentalis* and other thrips harmful to vegetable and ornamental crops in Europe. Wageningen Agricultural University Papers 95(1): 1–42.

[30] Gachon C, Mingam A, Charrier B (2004) Real-time PCR: what relevance to plant studies ? Exp Bot 55: 1445–1454. 10.1093/jxb/erh181 15208338

[31] Wong ML, Medrano JF (2005) Real-time PCR for mRNA quantitation. Bio Techniques 39: 75–85. 10.2144/05391RV01 16060372

[32] Caldana C, Scheible WR, Mueller RB, Ruzicic S (2007) A quantitative RT-PCR platform for high-throughput expression profiling of 2500 rice transcription factors. Plant Methods. 3(1): 7. 10.1186/1746-4811-3-7 PMC1914063 17559651

[33] Boava LP, Laia ML, Jacob TR, Dabbas KM, Gonçalves J, et al (2010) Selection of endogenous genes for gene expression studies in Eucalyptus under biotic (*Puccinia psidii*) and abiotic (acibenzolar Smethyl) stresses using RT-qPCR. BMC Res Notes 3: 43. 10.1186/1756-0500-3-43 PMC2854107 20181283

[34] Scharlaken B, Graaf DC, Goossens K, Brunain M, Peelman LJ, et al (2008) Reference gene selection for insect expression studies using quantitative real-time PCR: the head of the honeybee, *Apis mellifera*, after a bacterial challenge. J Insect Sci 8: 33.

[35] Ponton F, Chapuis MP, Pernice M, Sword GA, Simpson SJ (2011) Evaluation of potential reference genes for reverse-transcription-qPCR studies of physiological responses in *Drosophila melanogaster* . J Insect Physiol 57: 840–850. 21435341 10.1016/j.jinsphys.2011.03.014

[36] Teng XL, Zhang Z, He GL, Yang LW, Li F (2012) Validation of reference genes for quantitative expression analysis by real-time RT-PCR in four lepidopteran insects. J Insect Sci. 12: 60. 10.1673/031.012.6001 PMC3481461 22938136

[37] Chapuis MP, Tohidi ED, Dodgson T, Blondin L, Ponton F, et al (2011) Assessment and validation of a suite of reverse transcription-quantitative PCR reference genes for analyses of density-dependent behavioural plasticity in the Australian plague locust. BMC Mol Biol 12(1): 7. 21324174 10.1186/1471-2199-12-7 PMC3048552

[38] Sinha DK, Smith CM (2014) Selection of reference genes for expression analysis in *Diuraphis noxia* (Hemiptera: Aphididae) fed on resistant and susceptible wheat plants. Sci Rep 4. 10.1038/srep05059 PMC4034006 24862828

[39] Cheng D, Zhang Z, He X, Liang G (2013) Validation of reference genes in *Solenopsis invicta* in different developmental stages, castes and tissues. PLoS ONE 8(2): e57718. 23469057 10.1371/journal.pone.0057718 PMC3585193

[40] Fu W, Xie W, Zhang Z, Wang S, Wu Q, et al (2013) Exploring valid reference genes for quantitative real-time PCR analysis in *Plutella xylostella* (Lepidoptera: Plutellidae). International J Biol Sci 9(8): 792. 10.7150/ijbs.5862 PMC3753443 23983612

[41] Yang Q, Li Z, Cao J, Zhang S, Zhang H, et al (2014) Selection and assessment of reference genes for quantitative PCR normalization in migratory locust *Locusta migratoria* (Orthoptera: Acrididae). PLoS ONE 9(6): e98164. 24887329 10.1371/journal.pone.0098164 PMC4041718

[42] Shen Y, Gu J, Huang LH, Zheng SC, Liu L, et al (2011) Cloning and expression analysis of six small heat shock protein genes in the common cutworm, *Spodoptera litura* . J Insect Physiol 57: 908–914. 21510953 10.1016/j.jinsphys.2011.03.026

[43] Lu MX, Hua J, Cui YD, Du YZ (2014) Five small heat shock protein genes from *Chilo suppressalis*: characteristics of gene, genomic organization, structural analysis, and transcription profiles. Cell Stress Chaperon 19(1): 91–104. 10.1007/s12192-013-0437-8 PMC3857428 23702967

